# Rational bioinformatic approach to the analysis of functional properties of metabolites of probiotic microorganisms based on gene network reconstruction

**DOI:** 10.18699/vjgb-26-11

**Published:** 2026-03

**Authors:** V.A. Ivanisenko, T.M. Khlebodarova, M.A. Kleshchev, A.R. Volyanskaya, I.V. Yatsyk, A.V. Adamovskaya, T.V. Ivanisenko, P.S. Demenkov, N.A. Kolchanov

**Affiliations:** Kurchatov Genomic Center of ICG SB RAS, Novosibirsk, Russia; Kurchatov Genomic Center of ICG SB RAS, Novosibirsk, Russia; Kurchatov Genomic Center of ICG SB RAS, Novosibirsk, Russia; Kurchatov Genomic Center of ICG SB RAS, Novosibirsk, Russia; Kurchatov Genomic Center of ICG SB RAS, Novosibirsk, Russia; Kurchatov Genomic Center of ICG SB RAS, Novosibirsk, Russia; Kurchatov Genomic Center of ICG SB RAS, Novosibirsk, Russia; Kurchatov Genomic Center of ICG SB RAS, Novosibirsk, Russia; Kurchatov Genomic Center of ICG SB RAS, Novosibirsk, Russia

**Keywords:** industrial microbiology, functional microorganisms, probiotics, producer strains, metabolites, gene networks, ANDSystem, индустриальная микробиология, функциональные микроорганизмы, пробиотики, штаммы-продуценты, метаболиты, генные сети, ANDSystem

## Abstract

An important direction in industrial microbiology is the development of probiotic strains with valuable consumer properties. The probiotic industry is currently one of the most rapidly developing segments of the food and pharmaceutical sectors. Stearic (octadecanoic) acid C18:0 is one of the major metabolites present in the cell-free supernatant of the bacterium Streptococcus thermophilus, which is widely used in the production of fermented dairy products, including yogurt and cheese. S. thermophilus affects not only the texture and sensory properties of products, but also exhibits various probiotic effects, including antioxidant activity, modulation of the gut microbiota, inhibition of certain pathogens, and others. It is assumed that a number of probiotic effects exerted by S. thermophilus may be mediated through octadecanoic acid as one of its main metabolites. Octadecanoic acid C18:0, like other long-chain fatty acids, enters the human body via several mechanisms, including protein-mediated transport and passive diffusion across cell membranes. Inside the cell, octadecanoic acid serves not only as a substrate for the synthesis of triglycerides and other complex lipids, but, as shown in cell-based and in vivo models, also acts as a modulator of signaling and stress responses, including those associated with apoptosis. This is an important aspect of the influence of stearic acid on organism functioning, underpinning its anti-inflammatory and potentially anti-tumor effects. However, the molecular genetic mechanisms by which octadecanoic acid acts as a probiotic on the human organism remain insufficiently understood. In the present study, using our previously developed information – software system ANDSystem (employing machine learning and artificial intelligence for automatic extraction of knowledge from scientific texts and databases), we reconstructed gene networks regulating the intrinsic (mitochondrial) and extrinsic (death receptor-mediated) apoptotic pathways in human cells under the influence of stearic (octadecanoic) acid. To search for metabolites produced by probiotic microorganisms that may have beneficial therapeutic properties, we propose an approach that combines gene network reconstruction with differential gene expression analysis. Using this approach, we show that octadecanoic acid produced by S. thermophilus can control the intrinsic and extrinsic apoptotic pathways primarily via regulation of PTGS2 expression; the results indicate that cyclooxygenase-2 is a key regulator mediating the effect of octadecanoic acid on apoptosis-related genes.

## Abstract (ru)

Важное направление промышленной микробиологии – создание штаммов пробиотиков, обладающих ценными потребительскими свойствами. Индустрия пробиотиков является одним из наиболее динамично развивающихся сегментов пищевой и фармацевтической промышленности. Стеариновая (октадекановая) кислота C18:0 – один из основных метаболитов в клеточно-свободном супернатанте бактерии Streptococcus thermophilus, широко используемой в производстве различных ферментированных молочнокислых продуктов, включая йогурт и сыр. S. thermophilus влияет не только на текстуру и вкусовые свойства продуктов, но и обладает различными пробиотическими эффектами, в том числе антиоксидантной активностью, модуляцией кишечной микробиоты, ингибированием определенных патогенов и др. Предполагается, что ряд пробиотических эффектов, которыми обладает S. thermophilus, может быть опосредован именно через октадекановую кислоту, как основной метаболит. Октадекановая кислота C18:0, как и другие длинноцепочечные жирные кислоты, поступает в организм человека с использованием различных механизмов белок-опосредованного транспорта и пассивной диффузии через мембрану клеток. В клетке стеариновая кислота не только служит субстратом для синтеза триглицеридов и других сложных липидов, но и, как показано на клеточных и in vivo моделях, является модулятором сигнальных и стресс-ответов, связанных в том числе с апоптозом. Это один из важных аспектов влияния стеариновой кислоты на функционирование организма, определяющий противовоспалительный и потенциально противоопухолевый эффекты. Однако молекулярно-генетические механизмы влияния октадекановой кислоты как пробиотика на организм человека в этом отношении остаются недостаточно изученными. В настоящем исследовании с помощью разработанной нами ранее информационно-программной системы ANDSystem, использующей методы машинного обучения и искусственного интеллекта и предназначенной для автоматического извлечения знаний из научных текстов и баз данных, были реконструированы генные сети регуляции внутреннего (митохондриального) и внешнего (индуцируемого рецепторами смерти) пути апоптоза клеток человека под влиянием стеариновой кислоты. Для поиска метаболитов, продуцируемых пробиотическими микроорганизмами, обладающих полезными терапевтическими свойствами, разработан новый поход, включающий реконструкцию генных сетей и анализ дифференциально экспрессирующихся генов. На его основе было показано, что стеариновая кислота, продуцируемая S. thermophilus, контролирует как внешний, так и внутренний пути апоптоза через регуляцию экспрессии гена PTGS2, кодирующего фермент циклооксигеназу-2. Полученные данные позволяют рассматривать циклооксигеназу-2 как один из центральных регуляторов, опосредующих влияние стеариновой кислоты на экспрессию генов апоптоза. В работе предложен рациональный биоинформатический подход к поиску новых штаммов, обладающих пробиотическим потенциалом, на основе оценки действия продуцируемых ими метаболитов на целевые биологические процессы в клетках человека через реконструкцию генных сетей.

## Introduction

An important direction in industrial microbiology is the development of probiotic strains with valuable consumer properties. The probiotic industry is one of the most dynamically developing segments of the food sector with a large global market. Probiotic microorganisms are widely used in the production of fermented foods, dietary supplements, and specialized foods; industrial strains are required not only to be safe and technologically suitable, but also to provide pronounced functional effects (immunomodulatory, anti-inflammatory, metabolically mediated, etc.) (Terpou et al., 2019; Lau, Quek, 2024; Grujović et al., 2025).

Streptococcus thermophilus is a streptococcal species used in industrial biotechnology for food fermentation, particularly in yogurt and cheese production. It affects the rate of acidification of dairy products, their texture, and sensory properties, and also exhibits a number of probiotic effects including antioxidant activity, modulation of the gut microbiota, inhibition of certain pathogens, and others, which makes it attractive for industrial use (Cui et al., 2016). In a recent study, strains of S. thermophilus isolated from homemade and commercial fermented dairy product dahi were analyzed using genomics and gas chromatography – mass spectrometry profiling of cell-free supernatants. It was shown that a long-chain fatty acid – octadecanoic (stearic) acid (C18:0) – is among the major components of the supernatant (Sudheer et al., 2025).

This observation suggests that some functional effects of S. thermophilus may be mediated by octadecanoic acid as one of its principal metabolites synthesized by this microorganism

Like other long-chain fatty acids, stearic acid can enter human cells via multiple mechanisms, including proteinmediated uptake and passive diffusion across membranes. Key proteins involved in uptake include the CD36 translocase (SR-B2), a master regulator of cellular fatty-acid homeostasis (Chen et al., 2022; Glatz et al., 2022), and members of the SLC27/FATP transporter family, which import fatty acids coupled to their acylation by long-chain acyl-CoA synthetases (Mashek et al., 2007; Anderson, Stahl, 2013). In vivo, stearic acid delivery to the membrane is ensured by its reversible binding to albumin (Kamp, Hamilton, 1992; Richieri et al., 1993; Richieri, Kleinfeld, 1995).In human cells, stearic acid not only serves as a substrate for the synthesis of triglycerides and other complex lipids (Paton, Ntambi, 2009; Minville-Walz et al., 2010; Houten et al., 2016), but also – as demonstrated in cell-based and in vivo models – acts as a modulator of signalling and stress responses associated with apoptosis, tumor cell proliferation, leukotoxicity, as well as pro-inflammatory responses of macrophages and microglia (Evans et al., 2009; Yang et al., 2020; Hung et al., 2023). A review (Shen X. et al., 2025) discusses in considerable detail the functions of this vital molecule, including its role in such pathological processes as cardiovascular diseases, diabetes development, and liver damage. According to current evidence, stearic acid can influence cellular function by interacting with the CD36 receptor on the plasma membrane, followed by modulation of intracellular signalling pathways associated with this receptor (Chen et al., 2022; Glatz et al., 2022). Moreover, stearic acid can exert regulatory effects on the expression of several genes – in particular, through modulation of microRNA activity (Shen X. et al., 2025). This may be characterised as an intracellular mode of action of octadecanoic acid. However, the molecular-genetic mechanisms by which stearic acid – as a probiotic – affects the human body remain insufficiently understood. One important aspect of its influence is the modulation of programmed cell death levels (Yang et al., 2020), which constitutes a factor determining anti-inflammatory and potentially anti-tumor effects.Among the possible approaches to investigate the mechanisms of the potential influence of metabolites produced by microorganisms on the functioning of human cells, reconstruction and analysis of gene networks can be employed. A gene network is a group of coordinately functioning genes that control the phenotypic traits of an organism (Kolchanov et al., 2013). Interactions between genes in a gene network occur via their primary and secondary products – RNA, proteins, and metabolites. Reconstruction of gene networks allows identifying specific molecular pathways in human cells whose functioning is altered under the influence of various factors, including metabolites. It also enables prediction of the molecular-genetic targets of their action and their impact on disease prevention or development (Saik et al., 2019; Bragina et al., 2023; Ivanisenko V. et al., 2024).In the present study, using the previously developed information– software system ANDSystem – which employs machine learning and artificial intelligence methods (Ivanisenko V. et al., 2015, 2019) – we reconstructed gene networks regulating the process of apoptosis in human cells under the influence of stearic (octadecanoic) acid present in the cellfree supernatant of S. thermophilus. Analysis of these gene networks revealed that stearic acid controls apoptosis primarily through the regulation of the expression of the PTGS2 gene, which encodes the enzyme cyclooxygenase-2. An additional analysis of differential gene expression in HepG2 cell culture (Vendel Nielsen et al., 2013) under exposure to stearic acid showed that the differentially expressed genes were incorporated into the reconstructed gene networks.

The obtained results establish a rational bioinformatic approach to assessing the functional effects of microbial metabolites on target biological processes in human cells through gene network reconstruction. This approach can be applied in the search for new strains with probiotic potential.

## Materials and methods

**Reconstruction and analysis of gene networks **regulating human gene expression under the influence of stearic acid were carried out using the information-software system ANDSystem (Ivanisenko V. et al., 2019, 2024; Ivanisenko T. et al., 2020). ANDSystem is designed for the reconstruction of associative gene networks, and automatically extracts knowledge and facts from scientific publications and biological databases (Ivanisenko V. et al., 2015, 2019). One of the key modules of ANDSystem is a continuously updated knowledge base. At present, this knowledge base contains information on more than 150 million interactions between 12 different types of molecular-genetic entities (genes, RNA, proteins, metabolites, pharmaceutical compounds, etc.). This information has been automatically extracted from the texts of over 30 million scientific publications and patents, as well as from factual databases. The knowledge base encompasses 49 types of interactions between entities, including regulation of gene expression, physical interactions (protein–protein, protein–ligand), chemical interactions (catalytic reactions, post-translational modifications), and other interaction types (Ivanisenko T. et al., 2022). The effectiveness of ANDSystem for studying the molecular mechanisms of diseases, the effects of drugs and metabolites – including through the analysis of omics data (such as metabolomic and transcriptomic datasets) – has been demonstrated in numerous studies. For instance, it has been used for prioritization of apoptosis-related genes in lymphedema (Saik et al., 2019), identification of apoptosis genes as a basis for the comorbidity of Huntington’s disease and cancer (Bragina et al., 2023), and detection of metabolic markers of postoperative delirium (Ivanisenko V. et al., 2024).

**Analysis of differential gene expression and functional annotation.**To study the effect of stearic acid on gene expression, we analysed available transcriptomic data obtained using DNA microarray technology in an experimental study (Vendel Nielsen et al., 2013) that investigated the impact of fatty acids (elaidic, oleic, and stearic) on the metabolism of HepG2 cell culture (study identifier in the Gene Expression Omnibus (GEO) database: GSE34045). To date, this is the only experimental study that includes an analysis of the transcriptomic profile of cells exposed to stearic acid. Identification of differentially expressed genes (DEGs) was performed using the Limma package (Ritchie et al., 2015). The statistical significance of differences in gene expression levels between the control and stearic acid-treated samples was assessed using the false discovery rate (FDR) method. Differences with FDR values of less than 0.05 were considered statistically significant.Enrichment analysis of biological processes for the list of DEGs identified as described above was carried out using the DAVID web server, version 2021 (https://david.ncifcrf. gov/; Sherman et al., 2022) with default settings. DAVID evaluates the statistical significance of the overlap between the list of genes under study (in our case, DEGs) and gene lists corresponding to biological processes described in the Gene Ontology (GO) (Sherman et al., 2022).

## Results and discussion

**Reconstruction of regulatory gene networks of apoptosis controlled by octadecanoic acid**

As noted above, stearic acid can affect cell functioning both through the CD36 receptor on the plasma membrane (Chen et al., 2022; Glatz et al., 2022) and by penetrating into the cell and influencing gene expression inside it (Shen X. et al., 2025). In this context, based on the information contained in the knowledge base of the ANDSystem information-software platform, two gene networks regulating apoptosis induced by octadecanoic acid were reconstructed: an intracellular pathway of stearic acid action that does not involve the CD36 receptor protein, according to the ANDSystem knowledge base (gene network GN-VPDO), and a CD36-mediated pathway (gene network GN-CD36).

First, a list of human protein-coding genes involved in the apoptosis process was compiled – 748 genes obtained from the Gene Ontology database (https://geneontology.org/) for the term GO:0006915 (apoptotic process) (Supplementary Table S1)1. This list was used as input data in ANDSystem to reconstruct gene networks

Supplementary Materials are available in the online version of the paper: https://vavilov.elpub.ru/jour/manager/files/Suppl_Ivanis_Engl_30_1.zip


**Fig. 1.**
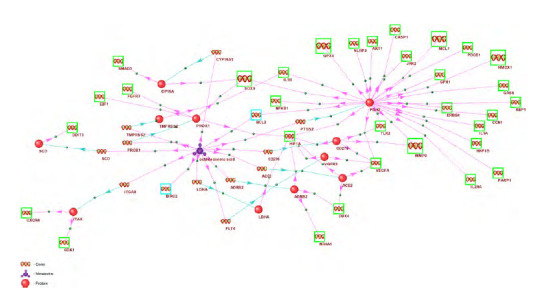
The GN-VPDO gene network representing the regulation of apoptosis-related gene expression by stearic (octadecanoic) acid. Green boxes indicate genes involved in the apoptosis process according to the Gene Ontology (GO:0006915, apoptotic process). Turquoise boxes mark apoptosis genes that are directly regulated by octadecanoic acid without intermediaries. Genes without boxes are intermediary genes: their expression is regulated by octadecanoic acid, and their protein products (red spheres in the figure) regulate the expression of apoptosis genes. Large boxes highlight genes that are differentially expressed in the HepG2 cell culture in response to octadecanoic acid (Vendel Nielsen et al., 2013). Stearic (octadecanoic) acid is labelled as “octadecanoic acid”. Blue arrows represent gene expression processes leading to the production of their encoded proteins; red arrows indicate the regulatory influence of proteins on the expression of apoptosis genes.

The gene network GN-VPDO, which represents the regulation of apoptosis-related gene expression by stearic (octadecanoic) acid entering the cell, is shown in Figure 1.

Analysis of the GN-VPDO gene network revealed that it included 33 apoptosis-related genes and an additional 11 genes involved in regulating their expression. The network also comprised 48 regulatory interactions between apoptosis genes and regulatory proteins, as well as 2 regulatory interactions between stearic (octadecanoic) acid and apoptosis genes (indicated by turquoise boxes in Figure 1).

**Fig. 2.**
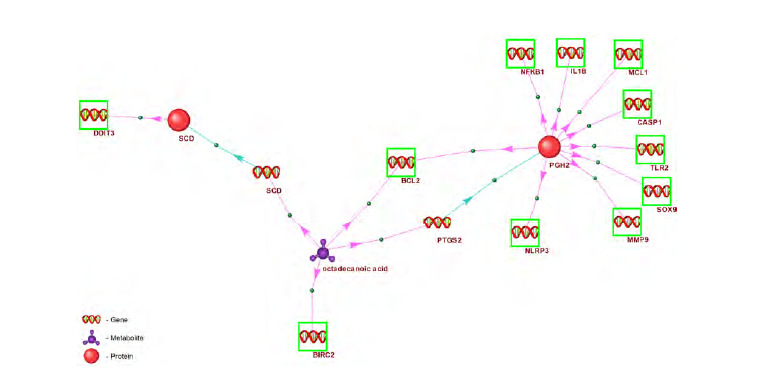
Fragments of the gene network showing regulatory interactions between stearic (octadecanoic) acid and the apoptosis-related genes BIRC2, SCD, BCL2, NFKB1, NLRP3, CASP1, and MCL1. Red spheres represent proteins encoded by intermediary genes. Green boxes indicate apoptosis genes. Genes without boxes are intermediary genes: their expression is regulated by octadecanoic acid, and their protein products (red spheres in the figure) regulate the expression of apoptosis genes. Stearic (octadecanoic) acid is labelled as “octadecanoic acid”. Blue arrows denote gene expression processes leading to the production of their encoded proteins; red arrows illustrate the regulatory influence of proteins on the expression of apoptosis genes.

Cyclooxygenase-2 protein had the highest number of regulatory connections (25) with apoptosis genes in the GN-VPDO network (Table 1). Additionally, PROX1 and CD276 proteins can be considered key regulators of apoptosis genes in this network, each regulating five genes (Table 1).Several examples of regulatory pathways through which octadecanoic acid affects apoptosis gene expression in the GN-VPDO network are presented in Figure 2.

**Table 1.**
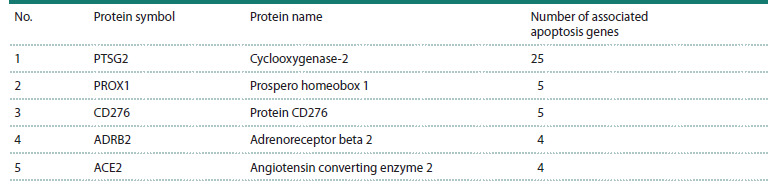
List of the five regulatory proteins with the highest number of regulatory connections to apoptosis-related genes in the GN-VPDO gene network

**Gene *BIRC2.*** Figure 2 shows that BIRC2 gene expression is under the regulatory influence of stearic acid. Suppression of this gene’s expression – which has anti-apoptotic activity – by stearic acid in preadipocytes represents one mechanism for reducing adipose tissue under the influence of this metabolite (Shen M.C. et al., 2014).

**Gene *SCD.*** According to ANDSystem data, stearic acid regulates the expression of the SCD gene, which encodes the SCD protein. This protein, in turn, regulates the DDIT3 gene, which is directly involved in apoptosis (Fig. 2). According to published literature (Aardema et al., 2017), stearic acid activates SCD gene expression. The SCD protein suppresses the DDIT3 gene (Minville-Walz et al., 2010), which is one of the key inducers of apoptosis (Bento et al., 2009).

**Gene *BCL2.*** The expression of the PTGS2 gene, which encodes the cyclooxygenase-2 protein (COX-2), is under the regulatory influence of stearic (octadecanoic) acid (Fig. 2). According to published literature (Liu J. et al., 2014), stearic acid induces the expression of the PTGS2 gene encoding cyclooxygenase- 2. In turn, cyclooxygenase-2 – as indicated by ANDSystem – enhances the expression of the BCL2 gene, a finding that is supported by experimental studies (Lin et al., 2019). It is worth noting that, according to both the ANDSystem knowledge base and published literature (Shen M.C. et al., 2014), the expression of the BCL2 gene can also be suppressed by stearic acid. Thus, according to gene network reconstruction data, the expression of the BCL2 gene – which encodes a key inhibitor of apoptosis (Newton et al., 2024) – can be both activated by stearic acid via cyclooxygenase-2 and suppressed by this metabolite, likely without the involvement of the enzyme. Therefore, modulation of either the expression or the activity of cyclooxygenase-2 may alter the balance between pro-apoptotic and anti-apoptotic effects of stearic acid. Available experimental evidence indicates that cyclooxygenase-2 inhibits apoptosis. Moreover, elevated expression of the cyclooxygenase- 2 gene is one of the mechanisms by which tumour cells evade apoptosis (Liu C.H. et al., 2001).

**Genes *NFKB1, NLRP3, CASP1, MCL1. ***According to the ANDSystem data (Fig. 2), the expression of NFKB1 and MCL1 genes – which encode the anti-apoptotic proteins NFKB1 and MCL1 (Newton et al., 2024) – as well as the NLRP3 and CASP1 genes encoding pro-apoptotic proteins (Yu et al., 2023), is regulated by stearic (octadecanoic) acid with the involvement of cyclooxygenase-2 (mentioned above). It has been demonstrated that cyclooxygenase-2 activates the expression of the anti-apoptotic genes NFKB1, MCL1, and BCL2 (Lin et al., 2019), which may account for the suppression of apoptosis by this enzyme

The GN-CD36 gene network of apoptosis regulation, reconstructed in our study, comprises 187 molecular-genetic entities, including 98 genes involved in the apoptosis process, the CD36 receptor protein itself, and 44 genes encoding proteins that exert regulatory effects on the expression of apoptosis-related genes. Due to the extensive number of regulatory interactions within the complete GN-CD36 gene network, it is presented in the Supplementary Materials (Fig. S1).

When analysing the complete GN-CD36 gene network (Fig. S1), several key regulatory proteins of apoptosis were identified: PPARG, which regulates 32apoptosis-related genes; cyclooxygenase-2, which regulates 25 genes; and SIRT1, which regulates 26 genes (Table 2).

**Table 2.**
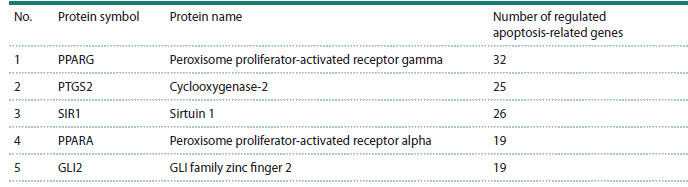
List of the five regulatory proteins with the highest number of regulatory connections to apoptosis-related genes in the GN-CD36 gene network

**Fig. 3.**
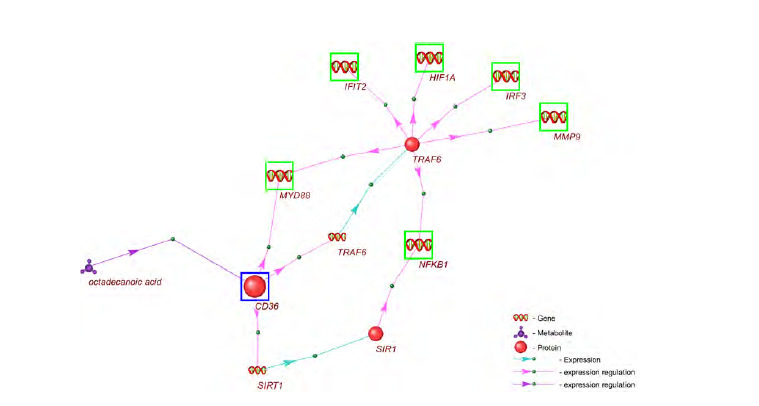
Fragments of the gene network showing regulatory interactions between the CD36 receptor and the apoptosisrelated genes IRF3, IFIT2, MMP9, HIF1A, and NFKB1 The CD36 receptor protein is highlighted with a blue box; apoptosis genes are indicated by green boxes. Genes without boxes are intermediary genes: their expression is regulated by octadecanoic acid, and their protein products (red spheres in the figure) regulate the expression of apoptosis genes. Stearic (octadecanoic) acid is labelled as “octadecanoic acid”. Blue arrows denote gene expression processes leading to the production of their encoded proteins; red arrows illustrate the regulatory influence of proteins on the expression of apoptosis genes. A purple arrow shows the interaction between octadecanoic acid and the CD36 receptor.

Figure 3 illustrates several examples of regulatory pathways through which the CD36 receptor influences the expression of apoptosis-related genes, as described below.

**Gene*NFKB1.*** Two regulatory pathways for the NFKB1 gene involved in apoptosis were identified. In the first pathway, CD36 regulates the expression of the intermediary gene SIRT1, which encodes the SIRT1 protein. SIRT1 then exerts a regulatory effect on the NFKB1 gene. According to (Yin et al., 2020), the SIRT1 protein has an activating effect on the NF-κB regulatory pathway. The second pathway involves the CD36 regulatory effect on the intermediary gene TRAF6 and its protein product, the TRAF6 transcription factor, which in turn regulates NFKB1 expression. The TRAF6 protein – whose gene expression is stimulated via CD36 (Cao et al., 2019) – also activates NFKB1 expression (Saba et al., 2014).

**Gene *MYD88.*** The CD36 receptor regulates the expression of the *MYD88 *gene, which is involved in apoptosis (a short regulatory pathway). Additionally, *MYD88* gene expression is regulated by the CD36 receptor through a longer pathway that involves the intermediary gene TRAF6 and its encoded protein, which exert a regulatory effect on *MYD88 *expression.

**Gene* MMP9.*** As noted above, the CD36 receptor protein regulates the expression of the TRAF6 gene, which encodes the TRAF6 protein. In turn, TRAF6 regulates the expression of the metalloproteinase gene *MMP9* (Luo et al., 2016), as well as five other apoptosis-related genes: IFIT2, HIF1A, IRF3, MYD88, and NFKB1. Thus, the TRAF6 transcription factor acts as a cassette activator of six apoptosis genes

A comparison of the GN-VPDO and GN-CD36 gene networks revealed that 30 genes involved in the apoptosis process were shared between these networks. Among them, the expression of 25 genes was regulated by cyclooxygenase-2 (Table S2). Consequently, this protein can be regarded as the most critical regulator of apoptosis genes in each of the two gene networks we reconstructed. The list of apoptosis-related genes common to both the GN-VPDO and GN-CD36 gene networks and regulated by cyclooxygenase-2 includes, among others, BCL2, MCL1, and NFKB1. The protein products of these genes are known to be key anti-apoptotic proteins (Gupta et al., 2023; Newton et al., 2024). On the other hand, the list of genes regulated by cyclooxygenase-2 in these gene networks also includes NLRP3 and CASP1, whose protein products are involved in activating programmed cell death processes, including apoptosis (Yu et al., 2023).

**Differential gene expression under exposure to octadecanoic acid**

To investigate the potential of stearic acid produced by the bacterium S. thermophilus in regulating apoptosis, we analysed experimental data on differential gene expression in hepatocyte-like HepG2 cells treated with stearic acid, as reported by L. Vendel Nielsen et al. (2013). We identified 2,500 differentially expressed genes (DEGs) with a statistical significance level of FDR < 0.05. A Gene Ontology (GO) enrichment analysis of this gene set revealed 40 statistically significant biological processes (FDR < 0.05) (Table S3), including cellular respiration, processes related to mitochondrial electron transport chain function, and apoptosis. The ten most statistically overrepresented biological processes for DEGs in HepG2 cells upon stearic acid treatment are presented in Table 3

**Table 3.**
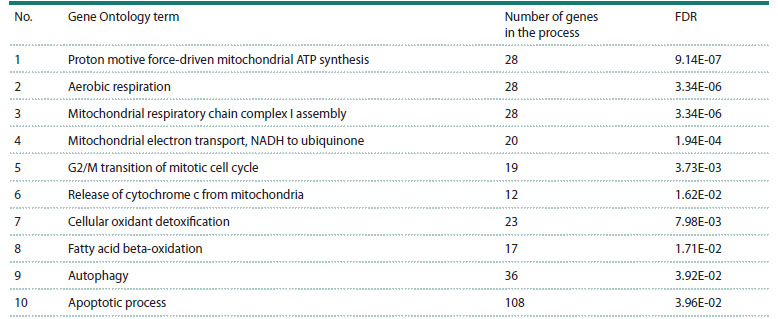
Results of Gene Ontology (GO) enrichment analysis for differentially expressed genes in HepG2 cell culture upon exposure to octadecanoic acid Note. FDR (false discovery rate) is a measure of statistical significance of biological process overrepresentation accounting for multiple comparisons.

A comparison of the reconstructed gene network for apoptosis-related gene expression regulation involving the CD36 receptor with differential expression data showed that among the apoptosis genes regulated by stearic acid, 16 genes (Table S4) exhibited statistically significant changes in expression in HepG2 cell culture upon exposure to the acid. These included genes such as MMP9, SOX9, HMOX1, GPX4, MCL1, and BIRC1, which play important roles in the apoptosis process. Regarding the GN-VPDO gene network, the number of apoptosis genes whose expression changed in response to octadecanoic acid was substantially lower: only five genes (GPX4, MCL1, HMOX1, MMP9, and SOX9). These same genes were also present in the GN-CD36 gene network. Thus, the analysis of differential gene expression indicates that the regulatory pathway via the CD36 receptor is more significant for stearic acid-mediated regulation of apoptosis

## Conclusion

In this study, we used the gene network reconstruction method to investigate the regulatory mechanisms by which stearic (octadecanoic) acid – one of the main metabolites in the cellfree supernatant of the bacterium S. thermophilus – affects apoptosis in human cells

We demonstrated that the GN-CD36 gene network described the regulation of 98 apoptosis-related genes, which significantly exceeded the number of apoptosis genes regulated in the GN-VPDO gene network (33 genes). Additionally, cyclooxygenase- 2 acted as the primary regulatory protein governing apoptosis gene expression under the influence of stearic acid, both in the GN-CD36 and GN-VPDO gene networks

**Fig. 4.**
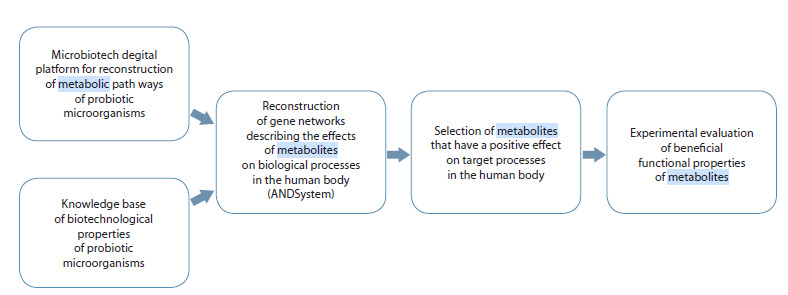
General scheme of the platform for identifying functional metabolites produced by probiotic microorganisms that exert beneficial effects on target processes in the human body.

These findings provide a foundation for developing an experimental-computational platform to identify functional metabolites produced by probiotic microorganisms that exert beneficial effects on target processes in the human body. The key components of this platform are presented in Figure 4.

The platform receives initial data from two sources. The first is the digital platform “Microbiotech”, developed within the framework of the Kurchatov Genomic Center at the Institute of Cytology and Genetics, Siberian Branch of the Russian Academy of Sciences (Demenkov et al., 2024). This platform enables the reconstruction of microbial metabolic pathways through computational analysis of their genomes. The second source is the Knowledge Base of Biotechnological Pro- perties of Microorganisms, which is being developed within the same Kurchatov Genomic Center using artificial intelligence methods. These methods allow for automatic extraction of knowledge and facts from scientific publications and patents.

The compiled data are used to reconstruct gene networks that describe the effects of metabolites on biological processes in the human body, employing ANDSystem (Ivanisenko V. et al., 2015, 2019). Analysis of the reconstructed gene networks helps to define selection criteria for metabolites that exert beneficial effects on target processes in the human body. In the final stage, an experimental assessment of the useful functional properties of the selected metabolites is carried out.

## Conflict of interest

The authors declare no conflict of interest.
